# Inflammation as an Animal Development Phenomenon

**DOI:** 10.1155/2012/983203

**Published:** 2011-10-19

**Authors:** Gustavo Campos Ramos

**Affiliations:** Department of Pharmacology, Biological Sciences Centre, Federal University of Santa Catarina, 88049-900 Florianópolis, SC, Brazil

## Abstract

Inflammation is a term that has been used throughout history in different contexts; it may represent a simple collection of clinical symptoms for which drugs are developed, a disease mechanism, or even a defense mechanism against microbes validating Pasteur's studies on bacteriology and Darwin's proposed struggle for survival. Thus, an explanation of this term must also consider the scientific questions addressed. In this study, I propose that several of the inflammatory events typically described in immunological, pathological, and pharmacological contexts can also be perceived as mechanisms of animal development. Thus, by recognizing that the generation of an animal form, its conservation, and its regeneration after tissue damage are phenomena of the same nature, inflammation can be addressed through the approach of developmental biology, thereby acquiring a much neglected physiological counterpart.

## 1. Introduction

The capacity to maintain and restore the integrity of tissues is crucial for the survival of all organisms, and the pathways through which the structure and organization of tissues are restored after a lesion may be as diverse as the forms of animal life. A starfish larva, for example, reacts to the insertion of a rose thorn with an intensive migration of phagocytic mesenchymal cells [1]. In contrast, an amphioxus responds to the same type of challenge through extracellular digestion promoted by the secretion of enzymes from its epithelial cells [2]. A salamander that has a limb amputated is capable of completely reconstructing a new functional limb, whereas other amphibians substitute the lost limb with fibrous tissue [3]. Even within a specific group (e.g., mammals), the pathways by which tissues are assembled and reassembled vary greatly. For example, a deep cut in the skin of an adult human generally triggers acute inflammation that is followed by a fibroproliferative process and scar formation; the same lesion inflicted on a fetus, however, may result in complete skin regeneration [4].

Irrespective of the peculiarities of the tissue repair processes in diverse groups of animals, it is rather intuitive to accept that regeneration and inflammation are related processes. Certainly, the processes that underlie the formation of a new salamander tail and the inflammation that occurs in response to a myocardial lesion in a mouse injected with high doses of isoproterenol are similar phenomena. Nonetheless, regeneration is viewed as the building of a structure, whereas inflammation is not recognized as such. These differences in our perception of these two phenomena are likely due to the history of the characterization of these phenomena, the experimental approaches used to address these topics.

## 2. Origins of Inflammatory Certainties

The initial framework used to study inflammation, which is widely accepted by the scientific community, is the description of the cardinal sinuses—*rubor et calor cum tumor et dolor*—performed by the Roman doctor Celsus approximately two thousand years ago [5–7]. This expression is still widely used and is representative of the general perception of inflammation. What is not usually mentioned, however, is that when Celsus proclaimed these words, he viewed inflammation simply as a collection of clinical symptoms and not as a phenomenon in itself. When Aristotle coined the term “development” to refer to the evolution of the shape of a chicken embryo, he defined it as a process of living. In contrast, inflammation put in Celsus terms was not considered to be a process, but rather it was defined as a collection of signals resulting from process which remain unknown. For a long time, pathologists studying inflammation contented themselves with enumerating more and more details of the inflammatory process in organisms becoming ill or injured tissues, without focusing on defining the concept of inflammation [8, 9]. 

As a result, in the 19th century, it was common to find scientists arguing for an end to the use of the term “inflammation” [10]. Even Virchow, the German pathologist who proposed a fifth cardinal inflammatory signal, *functio laesa*, categorically affirmed that inflammation was not a real entity [5], but a term that encompassed a series of phenomena so distinct that they should be treated separately.

From Celsus until the mid-19th century, the lack of an organismal/biological context to unify the different descriptions of the reactions of damaged tissue created a gap in the concept of inflammation. Even more concerning, the examination of this phenomenon, which was strictly medical, was isolated from other very similar biological processes. Animal regeneration, for example, was discovered in the 18th century and resulted in a period of enriching debates concerning the origin of the animal form [11]; during the same period, inflammation was reexamined through the lens of cell pathology. However, there was no attempt to converge these two ideas, as will be discussed later. The comparison of these two phenomena did not occur because regeneration was conceived as a phenomenon of form construction whereas inflammation had not attained the status of a biological phenomenon even by the end of the 19th century.

This perspective was changed by the seminal study described by Julius Cohnheim on the passage of white blood cells through capillaries and into inflamed tissue, known as diapedesis. For Cohnheim, this was not a mere histological description of what occurred in the disease process but the generative mechanism of the cardinal signs of inflammation. In describing changes in capillary structure, with the consequent movement of plasma and the passage of blood cells that compose the pus corpuscles, Cohnheim proposed a mechanism demonstrating how the symptoms described by Celsus were generated [12]. Thus, this proposal by Julius Cohnheim, demonstrating that the cause of inflammation resided in the vessels, unified diverse and subsidiary problems around a singular phenomenon and defined inflammation as an organic process. Within this context, inflammation was clearly a pathological event.

There is no doubt that this was a fundamental step in inflammation research. There are, however, two important limitations to Cohnheim's proposal. First, he suggested that inflammatory processes are exclusively pathological mechanisms without a physiological counterpart. Placing the vascular lesion as the ontological precedent of the inflammatory responses precludes the possibility of explaining how these processes are involved in the physiology of a healthy organism. This situation was unusual because we usually seek to understand the physiological role of a process prior to investigating its role in pathology. For example, we first sought to understand the electrical physiology of cardiac function prior to investigating the pathology of cardiac arrhythmias; however, we do not have the same reservations when studying inflammation in an exclusively pathological context. Except for very few instances, the term physiology is not mentioned in the immune-inflammatory jargon [13, 14].

A second limitation of Cohnheim's proposal on the origin of the cardinal signs of inflammation based on vascular events is that this process only occurs in a limited group of animals; only warm-blooded birds and mammals may present all the cardinal signs of inflammation. However, if the vascular lesion was a *sine qua non* condition for the emergence of an inflammatory dynamic, what would occur when an animal lacking a circulatory system suffered a tissue injury? How do all other animals repair themselves?

Therefore, although Cohnheim's explanation was a breakthrough we still lacked a broader perspective on the construction and reorganization of the organism. It was only when this problem was addressed by other disciplines that these limitations were noticed. Metchnikoff, a Russian embryologist, was important for this transformation.

Metchnikoff was interested in the formation of new embryonic forms during animal development and was investigating the role of a group of migratory and phagocytic mesenchymal cells in these phenomena. To Metchnikoff, understanding phagocytosis was important because this event can be observed in all animals, even in the simplest and most primitive forms (except in the amphioxus). When comparing phagocytosis among several groups of animals, Metchnikoff observed that phagocytes could ingest not only food particles, but also foreign particles and invading microorganisms (reviewed in [15]).

This last observation, in particular, acquired considerable relevance because it occurred at the time when Pasteur proposed that disease was caused by specific germs and when Darwin's work proposed the struggle for survival as the central problem in biology. With the proposal that phagocytes were a defense mechanism against the challenges of the environment, Metchnikoff united the most important medical and biological theories of the 19th century (1891), and because phagocytes are common to all animals, Metchnikoff understood that this would be the *primum movens* of inflammation. Thus, inflammation was transformed from a human pathological reaction to an animal health defense response [15].

It was Metchnikoff who developed the notion of a “defensive function” for inflammatory activity, which became fundamental to the modern concept of immunity. When Cohnheim described diapedesis, the defensive aspect was not included in his description. However, it is important to emphasize that Metchnikoff's idea of defensive action should not be accepted with naiveté, as is commonly the case when considering the notion of function. He had the insight to realize that the same process that the organism was using for defense was also participating in the embryonic and physiological processes of development. For example, Metchnikoff had already described that phagocytosis was involved in the reabsorption of the tail in one genus of amphibians. Thus, Metchnikoff described a physiological role for inflammation, and for him, the building of an organism was a problem that preceded its defense. For Metchnikoff, inflammation and immunity were subsidiary conditions of animal development; these situations were particular to the construction of metacellular harmony. Thus, he created the opportunity for studying the physiological aspects of inflammation [16].

Without discarding the important advances in pathology, Metchnikoff [1] circumvented the limitations of Cohnheim's proposal and developed an idea of great biological value. However, the only idea of Metchnikoff that was actually accepted by his peers was the defensive connotation of phagocytic activity (and generally with a naiveté that he himself lacked). All of his other considerations regarding the physiology of form construction in animals were quickly ignored. The newly founded discipline of immunology grew more concerned with understanding the pathogen-host relationship than any other generative or physiological aspect of the immune system or inflammatory activity. As a result, two main schools for addressing the inflammatory response were created: traditional pathology, which sees inflammation as a reaction to disease, and immunology, which sees it as a defense response.

A third more recent trend in inflammation research is the pharmacological approach. With the birth of the pharmacological industry, also at the end of the 19th century, the race to develop new methods to intervene in inflammatory processes became important. Thus, while pathologists described the reactions of organisms in response to disease, and the immunologists studied the detection of foreign bodies, the pharmacologists searched for methods to intervene in these events. In this context, there is one event in inflammation research that deserves to be highlighted: the invention of carrageenan-induced paw edema by researchers of the Merck pharmaceutical company [17].

During the first half of the 20th century, the methods for developing anti-inflammatory drugs were laborious and slow. In general, to characterize a potential anti-inflammatory agent, it was necessary to study inflammation as part of the repair of injured tissue. These models were tiresome, with protocols that lasted several weeks.

In 1962, a group of researchers from Merck developed a model that complied with all of the requirements needed by those interested in rapidly developing a product: carrageenan-induced paw edema in rodents. This protocol could be completed within four hours, it only needed one application of the drug to be tested, and its principle was based on the measurement of one of Celsus's cardinal signals: edema. For heuristic reasons, the industry created a model that separated the cardinal signals of inflammation from the complex processes of tissue repair; as a result, inflammation returned to being a mere clinical symptom.

The practical success of this idea was immediate, and in less than a year, Merck had developed indomethacin, a drug that still is used as a reference drug in the development of new anti-inflammatory agents. Other industries rapidly reproduced this model, and dozens of new anti-inflammatory agents successfully entered the market in that decade. It is interesting that this experimental protocol was widely accepted in the academic sphere and was widely used in basic research, as if it was an adequate model for understanding the inflammatory phenomenon. This had serious consequences because, by adopting a protocol that had been developed to simplify the research model for the pharmaceutical development of anti-inflammatory drugs, inflammation was once again no longer viewed as a physiological phenomenon; instead, it was studied as a cardinal sign of disease, as it had been two thousand years ago in Roman medicine.

## 3. Origin of Certainties in Regenerative Biology

The initial characterization of the regenerative process in animals is attributed to Adam Trembley for his studies on medusa polyps [11]. At that time, it was unclear if the medusa polyps were plants or animals. Thus Trembley sectioned them because only plants were thought to be capable of regeneration. Then after his experiment, it was observed that hydras had the capacity to reconstitute their lost parts, induce complete tissue repair, and rescue the *status quo ante* as if it was a plant. However, all other observations on the life cycle of these organisms led to the conclusion that they were indeed animals. Therefore, the possibility of animal regeneration was recognized with great surprise.

There is, however, something even more important in this finding by Trembley: because both halves of the hydra could promote a perfect tissue repair (regeneration), this situation was simultaneously a process of tissue reassembly and animal reproduction. Thus, the discovery of regeneration was also the discovery of a new form of asexual reproduction. This exceptional circumstance led to a conception of animal regeneration as not only a repair mechanism for lesions, but also as an event in animal development linked to the problem of reproduction and the generation of form. In addition, Trembley's finding occurred approximately at the same time as the climax of the embryological debate between preformationism and epigenesis in the 18th century. Therefore, it is not surprising that embryologists began to study regeneration [11].

Thus, contrary to inflammation, which in this period was barely treated as a phenomenon in its own right, animal regeneration emerged as part of a framework of well-defined biological ideas and occupied a central position in a world of rich debate. The theories of regeneration were placed next to those of embryonic development and metamorphosis; that is, regeneration was always perceived as a physiological phenomenon of animal development.

## 4. Inflammation as an Animal Development Phenomenon

Today, research in inflammation has certainly expanded its frontiers into several areas of scientific knowledge and could hardly be addressed within the limits of only one discipline. This plurality is not only desirable but also necessary. My particular interest in commenting on the emergence of the three main schools of inflammation research—pathological, immunological, and pharmacological—is not merely for the sake of providing historical background, but also to show that it is the context in which we make observations that defines the nature of the phenomenon being studied. Because of the way it has been perceived throughout history, inflammation emerged as both a symptom and a mechanism. I cannot negate the importance of the medical or industrial perspectives on this topic, but I hope to show that it is also valid to view the topic from a physiological and biological perspective. For this reason, I will not further discuss the definition of inflammation but show the delineations that it can be acquired when visualized within the context of developmental biology.

From the outset, one must perceive that although inflammation and regeneration are phenomena that emerge from very different needs, it makes no sense to study them separately within modern biology. Albeit legitimate, the medical interest in inflammation should not obscure the fact that, beyond the symptoms and magic bullets, inflammation can be seen in its physiological processes. Thus, there is a need to reconcile this theme with the development and construction of the animal form. More than their role in defense, inflammatory processes are part of the organism construction, and this can be illustrated with innumerable examples.

The urodele amphibians are capable of regenerating their eyes, including delicate tissues such as the retina and lenses. The surgical removal of the lenses from salamander newt eye triggers changes in the pigmented epithelial cells of the pupillary margin of the iris, which are capable of activating the cell cycle and differentiating into a new lens [18]. In a recently developed experimental model [19], it was shown that events typically described as immune inflammatory participate in the process of generating a new ocular lens. When the lenses are pricked with a needle through the cornea, they degenerate by autophagy, a process mediated by dendritic cells, and the elimination of the damaged lenses allows for the regeneration of new tissue from the dorsal edge of the iris. The authors observed that the transference of dendritic cells isolated from the ocular tissue of animals in the process of autophagy/regeneration into naïve animals (with their eyes untouched) was capable of promoting the genesis of a second lens even in the absence of injury. Furthermore, if the animals receiving a transplant of these activated dendritic cells were previously splenectomized, this generative process was inhibited. Therefore, the formation of new ocular tissues depends on processes that occur in a lymphatic organ, such as the spleen. This is an example of the generation of complex tissues mediated through immune-inflammatory processes; thus, this example illustrates the generative nature of inflammatory activity.

The proposal that immune-inflammatory activity is associated with generative phenomena gained even more experimental support when framed within the context of comparative evolution [20]. The tunicates (Urochordates) form a sister group of the vertebrates and thus occupy a relevant taxonomic position for understanding the phylogenetic origin of vertebrates and the adaptive immunity as well. In this context, the species *Botrylloides leachi* is a well-studied animal model for understanding the emergence of immunological activities. This species is a very common tunicate in the Mediterranean that exhibits the unique capacity of completely regenerating the adult organism from small vascular fragments. This phenomenon has been designated as whole-body regeneration. In a recent study, Rinkevich et al. [20] analyzed the complete mRNA profile transcribed during the process of full body regeneration and compared these profiles with other developmental processes such as metamorphosis, blastogenesis, and budding (asexual reproduction). To the authors' great surprise, they observed that

“comparison of genome-wide transcription of whole body regeneration with five other developmental processes in ascidians (including metamorphosis, budding and blastogenesis), revealed a broad conservation of immune signaling expressions, suggesting a ubiquitous route of harnessing immune-related genes within a broader range of tunicate developmental context.”

It becomes intuitive, after this revelation, that the problems addressed by embryologists and pathologists are phenomena of the same nature. However, we need to go further and recognize that the consequences of this admission are not trivial. The genesis of the biological form neither ends at birth nor resumes with disease. To understand this concept, it is necessary to view life as an incessant dynamic of transformation, like a Heraclitian fire [21]. To accomplish this, one must escape the animal models of birth and disease in which the framework is too well defined. In this respect, I believe that the hydras represent an interesting model to bridge the work of embryologists and pathologists.

## 5. Inflammation Physiology: Genesis Does Not End at Birth and Does Not Resume with Disease

An adult hydra polyp is comprised of two layers of epithelial cells: one derived from the endoderm and one derived from the ectoderm, which arrange to form a two-layer tube around the gastric cavity. At the apical end of this tube, there is a head where a mouth opening with tentacles is formed, and at the other end, there is a disc of cells responsible for attaching the organism to the substrate. In addition to these two layers, these animals are also constituted by a simple group of interstitial cells that give rise to neurons, gonads, and secretory cells [22]. 

**Figure 1:**
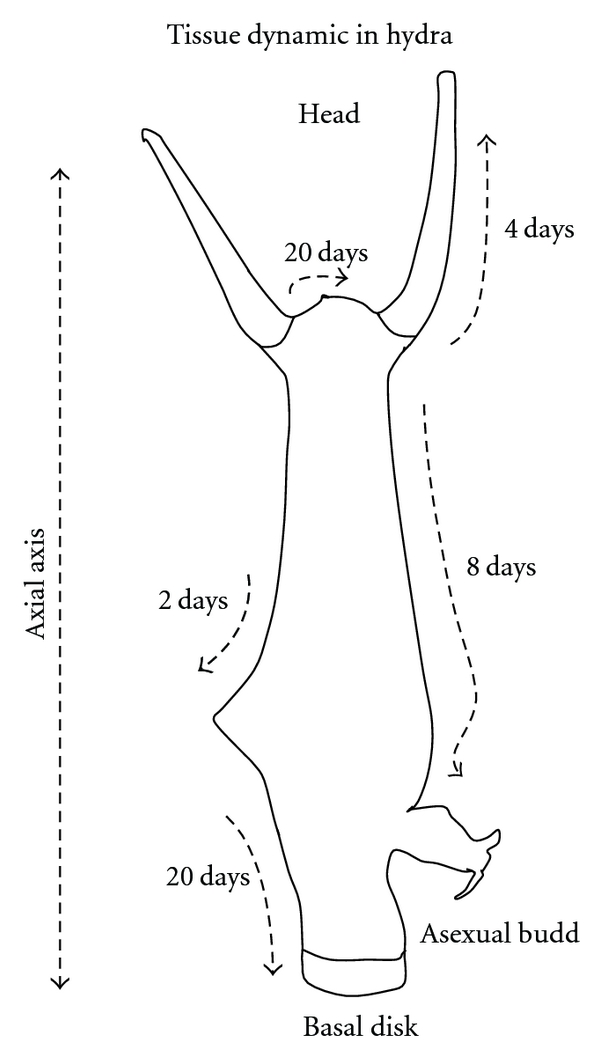
Tissue dynamic in a hydra polyp. Tissue movements were monitored after the insertion of tissue stained with methylene blue at different points, as described by Campbell [23]. The arrows indicate the direction and route of the tissue movements and the time elapsed. In these animals, cell identity is defined by its position relative to the axial axis, but these positions are not constant.

 In a series of elegant experiments, Campbell [23] stained the cells in several portions of this animal and tracked the dynamic movement of tissues during the life of the hydras. His findings were impressive because, although these animals conserve their body size for many years, the movement of tissues is incessant. Campbell [23] found that, given the incessant mitosis of the epithelial cells composing the hydra, all of the cells are constantly changing their position relative to the axial axis (Figure 1). It is a dramatic dynamic, although it is invisible to our eyes when we observe these animals *in natura*. It is also remarkable that, through these tissue movements, an epithelial cell that belongs to the central column of the organism will eventually reach the end of the body and will then differentiate into the specialized cells of the tentacles or basal disc [24]. Coordination of cell differentiation in the hydras depends on their position relative to the axial axis of the organism, but these positions are not constant. It is an incredible example of phenotypic plasticity that a cell with a given identity in a specific context can move to a new position and modify its phenotype. This same process occurs in the cells of the interstitial compartment. For example, a secretory interstitial cell found at a medial position along the anterior-posterior axis acquires a neuronal phenotype upon reaching the head [24]. The body is conserved, but all of its components continue changing and moving throughout its life.

The form of the animal is conserved throughout its life, but all of the mechanisms of generation and change are incessant. This example blurs the lines between embryology and pathology because, in this case, it is difficult to determine at which point “regeneration” has begun given that “generation” never ceased.

When we escape from animal models centered on the adult organism, we realize a second point that will be strongly defended in this paper: the genesis of form is never constrained to a particular moment in the animal's life cycle. The adult form is not finished, and it is not merely a product of our uterine past; instead, it is altered daily. Currently, this conserved generative process of daily life is neglected; it has not been studied by embryologists, whose study ends at birth, or by pathologists, who begin their study with a perturbation of the conserved shape. I believe that both disciplines have much to gain by extending their territories to study the condition of a healthy living adult. I believe this would have serious consequences because it would allow us to visualize a physiological counterpart of the studied processes that occur with disease—the physiology of inflammation.

A difficulty in accepting this proposal of the similarities between animal development and inflammation, which I call inflammation physiology, is our poor understanding of the living. An adequate view of the physiology of a healthy organism must include the tissue dynamic and the explicit notion that health, or physiological normalcy, is actively built. However, it is not just the medical realm that lacks a clear view of the organism; biology is also missing this view. Since Claude Bernard, we have greatly praised the notion of homeostasis, which is defined by a state of equilibrium. But this idea of a constant state is derived from an adultocentric premise that harmony between the parts needs no explanation.

In contrast, Metchnikoff, who came from the embryological world, perceived the complexity of the construction of form and profoundly disagreed with Bernard [16, 25]. For him, at the beginning of ontogeny there was disharmony; as a result, harmony was finely built and constantly woven. He was therefore interested in the construction processes. Moreover, because Metchnikoff studied phagocytes not only in their defensive context, but also in physiological situations, such as the metamorphosis of the tadpole's tail, he attributed the genesis of harmony to a “physiological inflammation” [15]; pathological inflammation was secondary to this incessant physiological genesis. In addition to Metchnikoff [1, 15], rare exceptions to a disease-biased view of the immune-inflammatory phenomena may also be found in the work of Vaz et al. (“conservative physiology of the immune system” [14]) and Cohen (body maintenance and “corrective inflammation” [26]).

Based on what has been described previously, I maintain that the corollary of this essay can be summarized as follows: *the generation, conservation, and regeneration of form* are all related problems, dealing with a more central question in biology, which is the construction of an organism. 

**Figure 2:**
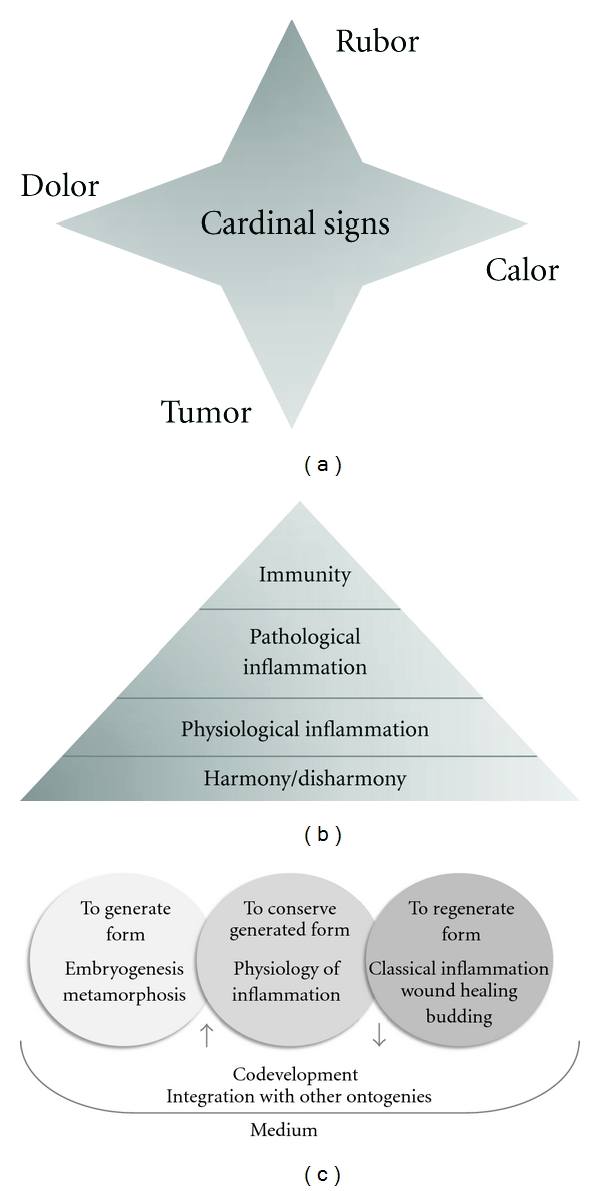
Inflammation in the eye of the observer. Since its origins, inflammation has frequently been viewed as a collection of *signals* (a). In this context, what is studied is not a biological process but the results of a process that remains ignored. According to Metchnikoff, however, inflammation and immunity are particular instances of a broader *process* of animal harmony generation ((b), adapted from Tauber [37]). Because the same mechanisms involved in pathology were also involved in embryogenesis, Metchnikoff coined the term physiological inflammation, which he claimed preceded the problem of pathological inflammation. A modern reinterpretation of this developmental contextualization of inflammation could be represented as in (c). Thus, the genesis of form, its conservation, and its regeneration are problems of the same nature, which deal with the construction of an organism even when examining the animal/microbiota interaction.

Currently, the term “inflammation physiology” has also been mentioned in the work of Medzhitov [27]. However, it is important to stress that the physiological aspects of inflammatory activity mentioned in the present paper and those mentioned by Medzhitov are concepts which arise from different questions; they are not different answers to the same question. Medzhitov's initiative is not meant to be a conciliation between inflammation and developmental biology nor a contemplation of the healthy living dynamics of self-construction. Thus, in his work the physiological inflammation is still perceived as a “response” to a stimulus. A schematic representation of the argument herein put forward, including a comparison to other schools of thought on inflammation, is presented in Figure 2.

## 6. Codevelopment: The Relationship with the Microbial World Revisited

Initially, an attempt to contextualize the study of inflammation within a developmental biology perspective seems to negate the main concern of this discipline, which is the defense against microbes. Clearly, we cannot neglect the importance of microorganisms in our lives or the fact that serious microbial infections exist. However, it is important to perceive that our current microbiological knowledge is much different from that which led Pasteur to propose the idea that germs cause disease. With the advent of molecular techniques for gene amplification, it was discovered, for example, that less than 1% of marine microbes grow on conventional culture media. Therefore, it was not possible to discover these microbes until very recently [28]. Thus, our understanding of microbial diversity has expanded at least 100-fold in recent years. In addition, it is worth highlighting the findings from a recent genomic characterization of the microbiota associated with humans, which discovered the existence of more than 2000 species of commensal microorganisms, of which less than 100 species are characteristically pathogenic [29].

Despite the importance of understanding the pathogenesis that emerges from the relationships among organisms, is it critical to recognize that microbial colonization is not a synonym of infectious disease (we are all healthy carriers of an immense diversity of microorganisms) and that a whole discipline exclusively dedicated to explaining infectious disease is a discipline that is focused on an exception [29]. A modern treatment of the relationship between microorganisms and their hosts should be capable of explaining both, medical bacteriology and also the recent explosion in our knowledge of microbial ecology.

In this context, one of the most renowned developmental biologist of our times, Scott Gilbert, has studied the integration of animal development within an evolutionary and ecological context (Eco-Evo-Devo), attributing great importance to the microorganism immune system interface in the process of constructing the animal form. Today, this is one of the most important and accepted perspectives in modern developmental biology, and it has as its main premise the idea that “all development is codevelopment.” Thus, in embryology, it is currently understood that it is not enough to reveal the details concerning the gastrulation of an animal, but it is also necessary to understand how its ontogeny is integrated with the ontogenies of its surrounding organisms [30]. In this codevelopmental context the host-microbial relationship and the immune-inflammatory phenomena has been revisited. According to some authors, such as Gilbert and Epel [30], Hooper and Gordon [31], and McFall-Ngai [29], who are important references in this field, immune-inflammatory activity can be viewed as a relevant phenomenon in the integration of the ontogenies of different organisms and not merely as a military defense system.

The host associations with bacteria, which codevelop with other organisms, have very curious nuances. The McFall-Ngai group studies the symbiotic association of certain squid species with bioluminescent bacteria (*Vibrio fischeri*) [32, 33]. These squids, which are nocturnal predators, are born without the bacteria and develop a rudimentary organ that hosts them and is an important step in their predatory behavior. This organ fully develops only in the presence of the bacteria, and these bacteria change their phenotype within the organ. It is also interesting to note that the components involved in the establishment of the relationships between these two organisms are exactly the same as those that participate in the reactions that we would consider to be inflammatory; peptidoglycans, Toll-type receptors, phagocytes, and nitric oxide synthase are involved in this process within a codevelopmental context.

This example has been frequently referenced as representative of this relationship. When we consider the mice with which we work, we return to the same warfare metaphors—defensive mechanisms—to describe inflammation. However, this is unnecessary. For example, the development of the vascular network in the intestinal villi of mice raised in the absence of bacteria (germfree) is severely damaged [34]. Thus, this certainly represents a codevelopmental issue.

The point is not to negate the occurrence of pathologies in relationships with intestinal bacteria, but rather to view it in terms of developmental deviations. We are not simply organisms that live to defend ourselves from pathogenic germs, as it was originally conceived when the concept of bacteriology was emerging with Pasteur. Today, we know that germ-free rats that receive an active transfer of adoptive microbiota from zebrafish, for example, assemble their native microbiota with the profile of their own species and not with the profile initially transferred from the zebrafish [35]. Similarly, the transfer of microbiota from rats into germ-free zebrafish generates the standard microbiota in the receiving fish species. Therefore, the relationships between the organism and its microbiota are actively built, and the microorganisms that we carry within us are not merely temporary passengers but carefully cultivated coinhabitants. Furthermore, in a series of elegant experiments comparing the gut colonization dynamic in gnotobiotic/Rag1^−^/^−^ and gnotobiotic/Rag1^−^/^−^ mice which received adoptive transfer with igA-producing hybridoma cells, Peterson et al. [36] have demonstrated that gut IgA promotes symbiosis homeostasis rather than immunological defense.

The formation of n bacteria biofilm in a particular region of the mammals' digestive tract is as sophisticated an event as the formation of a neuronal network in the encephalic region. Thus, it is possible to envision how immune-inflammatory activity plays a role in the construction of life even if it is involved in the establishment of relationships with other organisms.

## 7. Coda

The term inflammation has many different meanings depending on the context in which it is studied. Sometimes, it is defined as a salutary defense phenomenon; occasionally it is described as a pathological phenomenon, and on some occasions it does not even acquire the status of a phenomenon, being merely designated as a group of signals resulting from an otherwise ignored process. However, when the context of birth, which encompasses the discipline of embryology, and the context of tissue damage, which belongs to the discipline of pathology, are transcended, it can be seen that these disciplines are actually dealing with very similar problems. Curiously, this approach has been widely recognized when dealing with the regeneration of a limb in amphibians or an arm in starfish, but not when dealing with repair of injured tissues in mammals.

Therefore, in this work, I argue in favor of a rediscovery of inflammation as a phenomenon that also involves the genesis of form, through the example of the study of animal regeneration. This initiative does not negate the important pathological findings contributed to date, but embraces them within a broader context that is more in agreement with modern biology. Finally, this approximation between pathology and embryology is responsible for creating a physiological basis for describing inflammatory phenomena, which is currently neglected. Thus, by becoming a physiological process, *inflammation* becomes *formation*.
